# Posterior Semicircular Canal Plugging Relieves Tumarkin’s Crisis in Ménière’s Disease Patients

**DOI:** 10.3390/audiolres14030036

**Published:** 2024-05-09

**Authors:** Francesco Comacchio, Anna Bordin, Valerio Maria Di Pasquale Fiasca, Barbara Bellemo, Paola Magnavita, Elena Fasanaro, Elisabetta Poletto

**Affiliations:** 1 Otorhinolaryngology Unit, Regional Vertigo Specialized Center, Sant’Antonio Hospital, University Hospital of Padua, 35121 Padua, Italy; 2 Department of Neurosciences, Section of Otolaryngology, University Hospital of Padua, 35121 Padua, Italy

**Keywords:** Menière disease, semicircular canal plugging, Tumarkin crisis, Tumarkin syndrome

## Abstract

(1) Background: Patients affected by Ménière’s disease can experience Tumarkin’s syndrome, which is characterized by postural instability, gait abnormalities, and, occasionally, an abrupt loss of balance known as vestibular drop attack or Tumarkin’s crisis. In this study, semicircular canal plugging is proposed as the definitive treatment for this condition. The outcomes of this type of surgery are discussed. (2) Methods: A total of 9 patients with a confirmed diagnosis of Ménière disease suffering from Tumarkin crisis underwent posterior semicircular canal plugging. These patients were assessed with Video Head Impulse Tests, vestibular evoked myogenic potentials, and Pure Tone Audiometry preoperatively and postoperatively. (3) Results: VHIT showed a postoperative decrease in PSC gain median (Preop. 0.86 and postop. 0.52; *p* < 0.009). No statistically significant differences were described for the anterior semicircular canal and the lateral semicircular canal. No patient experienced new Tumarkin crisis after the surgical treatment. (4) Conclusions: Our ten years of experience with posterior semicircular canal plugging in Ménière disease patients with Tumarkin’s syndrome has shown that this type of surgical procedure is successful in controlling Tumarkin’s crisis, with high patient satisfaction and little worsening in hearing level.

## 1. Introduction

Ménière’s disease (MD) is an inner ear dysfunction that is characterized in its definite form by vertigo episodes lasting 20 min to 12 h [1], tinnitus, fullness in the affected ear, and initially fluctuating hearing loss that progresses over time. The origin of MD is related to the development of endolymphatic hydrops that can be visualized by magnetic resonance imaging [2]. Patients with MD may also experience postural instability, gait abnormalities, and, occasionally, an abrupt loss of balance known as vestibular drop attack or Tumarkin’s crisis (TC).

These dangerous and sometimes terrifying crises can also lead to severe traumatic injuries [3,4] and have, as main characteristics, a lack of warning signals and the absence of loss of consciousness. This last aspect was not confirmed by Pyykko et al. [5], who found that 12% of patients with MD reported in their case histories brief episodes of loss of consciousness, which were mainly related to TC in 4.7% of cases. 

The condition known as TC usually manifests in the latter stages of MD and was first reported by Tumarkin in 1936 [6] in a patient who fell to the ground unexpectedly without experiencing dizziness or other premonitory symptoms. Patients who have TC typically recover quickly and rapidly get up, without experiencing any aftereffects. However, many patients describe a lateral push sensation rather than a real drop attack. Another feeling that patients occasionally report is an abrupt loss of muscle tone in the lower extremities. Depending on which criterion is taken into account—as certain criteria for defining vestibular drop attacks may be more or less restrictive—the incidence of TC in MD patients has been reported in the literature to range from 3 to 72% [7,8] (for severe or mild forms of the phenomenon). We prefer the term Tumarkin crisis to vestibular drop attacks because, in our experience and as noted by Kuhl [9], the patients most frequently refer to a lateral pushing sensation rather than vertical falls like a drop, which are more typical in non-vestibular forms of drop attacks. The exact mechanism responsible of TC is currently unknown. Significant hydrop was documented in the vestibule and in the cochlea in all MD patients suffering with TC [10]. The pathophysiology of the crisis has been linked to sudden otolith dysfunction of the utriculus and/or the sacculus due to changes caused by the endolympathic hydrop. It has been also proposed that macular dysfunction in patients who lose consciousness during a TC may modify the sympathetic–vestibular reflex, which may result in orthostatic hypotension [5,11].

Ten years ago, we performed surgery on a woman with MD to plug her posterior semicircular canal (PSC). She had experienced multiple episodes of benign paroxysmal vertigo brought on by PSC canalolithiasis, as well as TC episodes that resulted in serious head trauma and arm fractures. Throughout the extensive follow-up, the patient never had TC. Subsequently, we subjected another eight patients with MD who were afflicted with TC to PSC plugging, and in every case, TC was completely relieved. The best surgical technique for intractable BPPV is currently thought to be PSC plugging, which was first described by Parnes and McClure in 1990 [12], but it is rarely indicated [13,14]. However, its effect on TC has never been tested. This study examines the outcomes of this type of surgery, as well as the possible factors that contribute to the positive results. 

## 2. Materials and Methods

### 2.1. Study Design

The researchers used a retrospective design to analyze the charts of patients who underwent surgery for TC. Two visuo-analogic scale (VAS) questionnaires were administered during a recent checking visit. The Ethical Committee of the University Hospital of Padua gave its approval for this research. 

### 2.2. Participants

All adult patients treated by the senior author (FC) with surgical PSC plugging for MD with TC between January 2014 and November 2023 had their charts retrospectively reviewed. Individuals undergoing triple plugging were not included in the study. Individuals who had benign paroxysmal positional vertigo (BPPV) or posterior canal blockage for dehiscence alone were also excluded. Each patient’s medical history was checked to assess the presence of other conditions which could affect falls. No additional exclusion standards were used.

### 2.3. Data Collection

**Figure 1:**
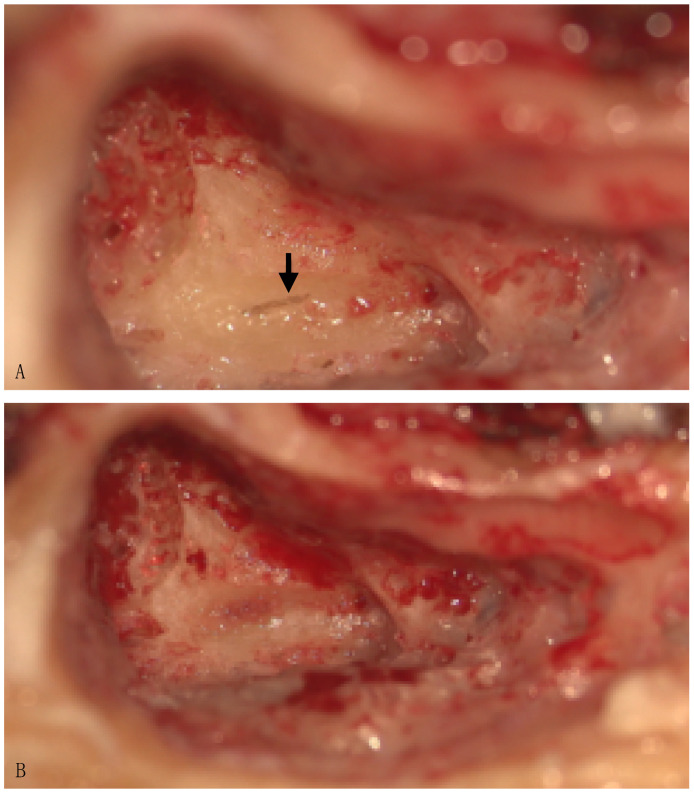
Right-ear PSC plugging. (**A**): PSC opening (arrow); (**B**) image showing the outcome following temporal muscle plugging.

All patients subjected to PSC plugging underwent brain magnetic resonance imaging (MRI) during the routine examination for MD and a preoperative ear and temporal bone CT scan. Baseline Pure Tone Audiometry was carried out in all patients and throughout the follow-up period. PTA for 0.5-1-2-4 Khz (PTA-4) was considered for statistical analysis. The vestibular assessment included a Video Head Impulse Test (VHIT) for all semicircular canals (ICS Impulse, Otometrics/Natus, Taastrup, Denmark, and EyeSeeCam, Interacoustics VisualEyes™, Middelfart, Denmark) and air-conducted cervical and air- and bone-conducted ocular vestibular evoked myogenic potentials (AC-CVEMPs and bc-oVEMPs) (Eclipse, Interacoustic) before surgical treatment and after the surgery. cVEMPs were elicited using a 500 Hz tone burst, with 4.0 msec. of rise–fall time and plateau. The tone bursts were presented monaurally via headphones (Telephonics TDH-49) at dB HL, at a repetition rate of 5.1 bursts/s. EMG activity was amplified and bandpass filtered between 10 and 1500 Hz. At least 200 cVEMP responses were obtained and averaged for each recording. The same stimulus, with 0.1–1000 Hz filtering, was delivered via a radio-Ear B71 bone transducer for oVEMP recording. Two VAS questionnaires, ranging from 0 to 10, were administered to the patients by phone contact. One investigated the presence of an improvement in global quality of life (QoL) after the surgery, and the second, regarded the specific item of QoL related to the presence of falls. A score of “0” represented the absence of change, while “10” represented a highly satisfactory change. PSC plugging was performed in all patients with a transmastoid approach, the exposition of the labyrinthine block, and the visualization of the blue line of the PSC and its opening (Figure 1) without injuring the membranous labyrinth. A piece of temporal muscle was introduced in the canal, and bone wax was used as a sealant.

### 2.4. Statistical Analysis

A descriptive analysis of the recorded data was performed. The qualitative variables are reported as frequencies and percentages, and the quantitative variables are summarized as medians and interquartile ranges (P25–P75) and means and standard deviations. The normality of the variables’ distribution was assessed via a Shapiro–Wilk test and graphically with QQ-plots. A Wilcoxon test for paired samples was applied to evaluate possible differences between preoperative and postoperative assessments of vestibular and hearing function in the preoperative and postoperative phases. The significance level was set at 5%, and a 95% confidence interval was considered. Statistical analysis was performed using R software (version 4.3.2).

## 3. Results

**Figure 2:**
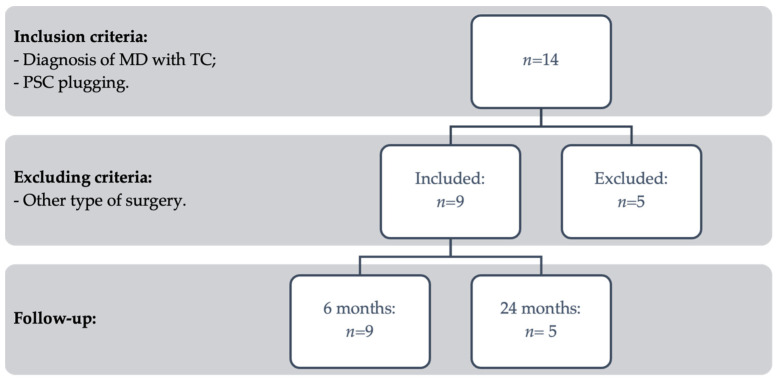
Flow chart describing case inclusion process.

In this study, nine out of 14 patients who underwent canal blocking surgery for MD and TC were taken into account. The inclusion process is reported in Figure 2. Five individuals who underwent a triple plugging treatment were eliminated. Seven men and two woman made up the patient group; their ages ranged from 56 to 78 years old, with a mean age of 68.78 ± 7.71 years. Every patient had unilaterally defined MD. No presence of other conditions that could affect falls was detected in patients’ medical histories.

The patients’ epidemiological data are shown in Table 1. The condition lasted 12.00 ± 8.45 years, indicating that TC typically occurs at the final stages of the illness. Four patients had complex falls: one patient had a head trauma, another had a wrist fracture, and another patient had a homer fracture. The follow-up period after surgery was lengthy, ranging from 6 to 121 months, with a mean of 46.22 months. 

### 3.1. Vestibular Function

All of the data from the vestibular evaluation performed using VHIT both before and after surgery, as well as the examination of the ocular and cervical VEMPs, are presented in Table 2. Regarding PSC gain at VHIT, all patients presented a decrease in gain, with mean differences showing statistically significant differences (0.86 and 0.52; *p* < 0.009). The differences in the means before and after surgery for the anterior semicircular canal (ASC) and lateral semicircular canal (LSC) were not statistically significant. After examining the data, we can see from the table that only patient (n.1) experienced a reduction in PTA-4 at Pure Tone Audiometry and a general decrease in labyrinth function affecting all three semicircular canals. Another patient, number 8, showed a decrease in the PSC and LSC following surgery but not in the ASC. According to the table, every patient had a pre-op significant utricular impairment as evidenced by the BC-oVEMPs, which, in most cases, indicated a decrease in the amplitude of the N10 component or a lack of response in the afflicted ear. Two patients did not undergo the VEMP test. In our series, 50% of patients who conducted the test had normal amplitude P13-N23 potentials, suggesting that AC-cVEMPs may be more resistant to the disease. In contrast to the healthy ear, two patients displayed a reduced response, while two patients displayed no response at all. After surgery, the amplitude of the AC-cVEMPs decreased in only one patient. The remaining patients’ responses were unaffected by the procedure. 

### 3.2. Auditory Function

Pure Tone Audiometry, performed prior to surgery in our case series, revealed a PTA-4 of 63.61 +/− 17.37 dB, which is consistent with a long-term decline in hearing function (Table 3). Statistical analysis revealed that the mean postoperative PTA-4 did significantly deviate from the pre-op mean (63.61 +/− 17.37 vs. 73.61 +/− 17.31 dB, *p* < 0.034). The difference between the preoperative and postoperative PTA-4 means was 9.45 dB. In two cases, n.1 and n.6, a significant drop in the Pure Tone Audiometry threshold was noted (n = 25). After surgery, the second patient lost all of his auditory ability but had a significant pre-op PTA-4 threshold of 80 dB. The first patient had a loss of 30 dB of PTA-4. 

### 3.3. VAS Questionnaires

Table 4 shows the global results of the VAS questionnaires concerning an improvement in overall QoL and QoL related to the recurrences of falls after the surgical act. No patients experienced any falls after surgery, and the overall QoL indicated high levels of satisfaction.

## 4. Discussion

The most severe Meniere’s disease complication is TC. TC typically occurs in the latter stages of the illness, but it can also show up a few years after MD initially manifests; Baloh et al. [15] reported a patient whose TC was the first sign of MD. Older individuals with recently developed Meniere’s disease have a higher incidence of drop attacks, according to Ballester et al. [16]. This could be due to older people’s decreased otolithic structural compliance. Our cases have an average disease duration of 11 years, which confirms that most cases of TC occur in the late stages of the disease and primarily affect elderly patients [17]. However, our cases also support earlier data suggesting that TC may occur at the beginning of the disease in a small number of cases, as demonstrated by case n.9, an elderly patient who experienced three separate falls within months of each other after a few months of the classic initial audiovestibular symptoms. For the patients, the symptoms’ most upsetting aspects are their abruptness and lack of warning [18]. Patients almost always fear another episode and are alerted to the potential consequences of falling. According to Pyykko et al. [5], our data appear to support the theory that certain patients, as evidenced by patient n.10, may have experienced brief periods of LOC during the attacks. The same authors [5] reported that 14% of patients with MD have syncope as a result of otolith system failure in TC, most likely as a result of an incorrectly triggered vestibular sympathetic response. It is commonly recognized that TC typically manifests in a flurry that resolves in spontaneous remission [19]. The likelihood for spontaneous remission in TC has sparked significant debate on its course of treatment. Since medical treatment frequently does not work, Black et al. [20] suggested surgery. Recently, Carmona et al. [21] reported in their case series that most of their MD patients with TC showed an ineffective response to betahistine treatment. A cautious approach is advised by Baloh et al. [15] and Janzen et al. [18], who did not uncover many serious complications in their series. 

In addition, Pyykko et al. [3] discovered that 38 of the 92 individuals who had TC had bruising, 12 had fractures, and 3 had serious back injuries. Similarly to our patients, another individual suffered numerous injuries in a car accident. There has been a recent association between the phenotype of individuals with MD and traumatic brain traumas and frequent vestibular drop attacks (VDAs) [22]. According to Soto Varela et al. [23], TC significantly increases the severity of MD, and these patients frequently need psychological care [24]. Additionally, a very high percentage (50%) of complex fall events occurred in our series. This high rate could be explained by the fact that all of our patients wanted surgery to address their issues and prevent similar incidents in the future. 

Therefore, we believe that adopting a waiting attitude entails some medical responsibility. According to Kutlubaeva et al. [7] and Kaasinen et al. [25], patients often seek a potential decisive intervention in order to prevent having to live with the sword of Damocles looming over them. As demonstrated by earlier reports [3] and by patient n.9, these patients frequently avoid being away from home for extended periods of time after experiencing TC. These were the main causes of surgery in most of our instances. For the treatment of TC, a number of surgical techniques have been proposed. Intratympanic gentamicin has been a frequently used initial therapeutic option for TC, but it does not correspond with standard medical procedures, with different degrees of efficacy being reported [10,24,26]. Gentamicin, on the other hand, is said to control 84–87 percent of cases [10], although occasionally a destructive dosage is required to eradicate TC [27]. Labyrinthectomy has been reserved for cases with severe hearing loss and can completely abolish the auditory and vestibular function. Conversely, vestibular neurectomy preserves hearing and completely eliminates vestibular activity, but in rare cases, it may result in serious complications, and a small percentage of patients experience failure. Additionally, one case of cochlear implants being effective in relieving TC has been reported [28]. Recently, semicircular canal blocking was proposed as an effective means of managing refractory MD [29,30,31]. The advantage of plugging is that, in most situations, it retains the remaining vestibular organs and auditory function. In our series, only two patients exceeded the PTA threshold proposed by the guidelines for the evaluation of therapy in MD published by the American Academy of Otolaryngology—Head and Neck Surgery Committee on Hearing and Equilibrium [32]. According to Zhang et al. [30], who compared the triple plugging procedure with intratympanic gentamicin administration in controlling refractory MD, there was no appreciable difference in the rate of hearing loss between the two groups, but the rate of vertigo control with triple semicircular canal plugging was significantly higher than the rate with chemical labyrinthectomy. Our PSC plugging-based results appear to support the procedure’s effectiveness in preserving hearing. 

The most important finding in our research was the control of the Tumarkin crisis, which was achieved with PSC plugging. According to the findings of the VAS questionnaires, the patients are quite satisfied with the treatment’s efficacy, which indicates that the treatment is stable and long lasting. Several theories might be put out to account for these findings. 

The most accepted idea for explaining the pathophysiology of TC is associated with abrupt changes in the utriculus and/or sacculus’s otolith function as a result of altered inner ear pressure gradients [15] or unstable otolithic function [33]. Although MRI has shown isolated saccular hydrops in TC patients [34], a great deal of focus has been placed on utricular failure as the primary cause of TC [35]. 

Recently, Calzada et al. [36] found that all patients who experience TC show disrupted utricular otolithic membranes. In their study, all patients with TC demonstrated a disrupted utricular otolithic membrane in comparison to only the 50% and 56% of patients with delayed endolymphatic hydrops and Meniere’s disease without TC, respectively. Senofsky et al. [37] recently speculated, in a numerical model, that a disrupted otolithic membrane is, by itself, capable of generating abnormal spikes, thus being theoretically responsible for TC.

The results of the preoperative VEMP study in our patients documented a significant utricular pathology. No patient presented abnormal oVEMPs, while 40% showed normal cVEMPs. The contemporary lesion of the saccule during the surgical procedure might be one possible explanation for the positive results of PSC plugging, but in most cases, the cVEMP values were unchanged postoperation. This observation brought our attention to the utricle and the PSC. Except for patient n.1, who clearly presented global labyrinthine damage postoperatively, and those for which the abolished TC can be easily explained, all the other patients presented a significant reduction in the PSC function documented by VHIT. Thus, a potential connection between the PSC and the utricle in the development of TC is conceivable. A vestibular drop attack is not exclusive to MD patients. After the Epley maneuver, patients with BPPV may also experience sudden falls [38]. The abrupt displacement of otoliths from the PSC in the utricle has been linked to this event. Patients 1, 3, and 5 reported prior BPPV episodes during the MD course, suggesting that canaliths present in the PSC may be the cause of the aberrant utricle reaction in TC. An alternative hypothesis is that the PSC operation considerably altered the labyrinth’s pressure gradient and endolymphatic dynamic, preventing the formation of TC. 

Furthermore, we have to consider that the PSC is the main semicircular canal related to the lower spinal connection and that the integration between otolith apparatus and the semicircular canals is necessary for static and dynamic postural control [39].

It was proposed that the short-latency vestibulospinal reflex, which maintains postural stability, is linked to vestibulospinal neurons activated by the PSC, which are situated within the focal area of the vestibular nuclei and have strong connections with the lower segments of the spinal cord [40,41]. Lelonge et al. [4] observed a spontaneous vertical down-beating nystagmus immediately after TC that occurred in an MD patient in their waiting room. The authors hypothesized that the vertical semicircular canals had a part in the development of TC as a result of this observation. Based on all of these studies, it appears likely that the PSC, in particular, and the vertical semicircular canals may have an impact on how TC develops.

## 5. Conclusions

To sum up, our ten years of experience with PSC plugging in MD patients with TC has shown that this type of surgical procedure is successful in controlling falls, with high patient satisfaction and without significantly worsening hearing loss, and that the PSC is likely involved in the genesis of TC, along with the otolithic system. Due to the rare nature of this type of surgery, this study has limitations in that there are only a small number of patients in the series and vestibular assessment data for some patients who underwent surgery years ago are missing. This study’s main strength was its extensive follow-up. 

## Figures and Tables

**Table 1:** Demographic and general data of the analyzed sample.

#	Sex	Age (yrs)	Disease Duration (yrs)	Follow-Up (mos)	Complications
1	M	78	6	22	homer fracture
2	M	75	23	15	head trauma
3	M	58	25	49	
4	M	72	10	98	wrist fracture
5	M	76	19	54	
6	F	68	6	121	head trauma
7	M	66	6	34	
8	M	56	12	17	
9	F	70	1	6	
	7:2	70.00 (56.00–78.00) * 68.78 (7.71) ^+^	10.00 (1.00–25.00) * 12.00 (8.45) ^+^	34.00 (6.00–121.00) * 46.22 (39.56) ^+^	4 (44.44%) ^§^

* median (range); ^§^ N(%); ^+^ mean (SD).

**Table 2:** Preoperative and postoperative evaluation of vestibular function with Video Head Impulse Tests and ocular and cervical vestibular evoked myogenic potentials.

#	PSC Gain		LSC Gain		ASC Gain		cVEMPs		oVEMPs	
	Preop.	Postop.	Preop.	Postop.	Preop.	Postop.	Preop.	Postop.	Preop.	Postop.
1	0.83	0.29	0.70	0.39	0.75	0.33	nr	nr	nr	nr
2	0.71	0.38	0.83	0.78	0.70	0.58	n	n	ar	ar
3	0.78	0.32	0.90	0.83	0.75	0.70	ar	ar	ar	ar
4	0.70	0.36	0.80	0.83	0.82	0.78	nr	nr	nr	nr
5	0.65	0.37	0.92	0.93	0.90	1.10	n	n	ar	ar
6	0.78	0.56	1.24	1.20	0.88	0.85	a	a	a	a
7	0.88	0.52	1.00	1.05	0.85	0.85	n	ar	a	a
8	0.89	0.55	0.90	0.56	0.90	0.85	a	a	a	a
9	1.50	1.32	1.02	0.57	0.85	1.21	n	n	a	a

PSC: posterior semicircular canal; LSC: lateral semicircular canal; ASC: anterior semicircular canal; cVEMPs: cervical vestibular evoked myogenic potentials; oVEMPs: ocular vestibular evoked myogenic potentials; nr: not reported; n: normal; ar: amplitude reduction; a: absent.

**Table 3:** Preoperative and postoperative evaluation of hearing function with pure tone average.

#	PTA (dB)	
	Preop.	Postop.
1	37.5	68.75
2	73.75	72.5
3	61.25	68.75
4	72.5	80
5	71.25	75
6	80	110
7	75	75
8	70	70
9	33.75	40
	71.25 (33.75–80.00) * 63.89 (16.80) ^+^	72.50 (40.00–110.00) * 73.33 (17.89) ^+^

* median (range); ^+^ mean (SD). PTA: pure tone average.

**Table 4:** VAS questionnaires’ postoperative results.

#	VAS Questionnaires	
	VAS	VAS Falls
1	7	10
2	10	10
3	9	10
4	nr	nr
5	10	10
6	10	10
7	10	10
8	8	10
9	9	10
Mean	9.125	10

VAS: visuo-analogic scales; nr: not reported.

## Data Availability

No new data were created or analyzed in this study. Data sharing is not applicable to this article.
